# Overexpression of RAB34 associates with tumor aggressiveness and immune infiltration in glioma

**DOI:** 10.1042/BSR20212624

**Published:** 2022-10-28

**Authors:** Peng Hou, Quan Wan, Qing Wang, Xuechao Wu, Xiaojie Lu

**Affiliations:** 1 Department of Neurosurgery, The Affiliated Wuxi No. 2 People’s Hospital of Nanjing Medical University, Wuxi 214002, China; 2 Department of Neurosurgery, Nantong Hospital of Traditional Chinese Medicine, Nantong 226001, China; 3 Department of Neurosurgery, The Affiliated Wuxi Clinical College of Nantong University, Wuxi 214002, China

**Keywords:** EMT, glioma, immune checkpoint, immune infiltration, methylation, RAB34

## Abstract

RAB34 (RAB34, member RAS oncogene family) is aberrantly expressed in various cancers and exhibits oncogenic properties. However, its function in glioma remains largely unclear. In the present study, we collected 697 RNA-seq data from The Cancer Genome Atlas (TCGA) dataset and 325 RNA-seq data from Chinese Glioma Genome Atlas (CGGA) dataset. Bioinformatics and PCR analysis showed that RAB34 expression was positively related to the glioma tumor grade and predicted poor outcomes for glioma patients. Additionally, RAB34 expression was significantly up-regulated in classical and mesenchymal subtypes, and isolated diastolic hypertension wild-type gliomas. Moreover, RAB34 expression was remarkably correlated with inflammatory activities, immune infiltration, and immune checkpoints in glioma. *In vitro* experiments demonstrated that inhibition of RAB34 restrained the growth, migration, as well as invasion of glioma cells, and reversed the epithelial-to-mesenchymal transition (EMT) process. Our findings established RAB34 as a novel progression-related biomarker and a possible immunotherapy target for glioma.

## Introduction

Glioma is the most frequent primary brain tumor, exhibiting high morbidity, a low rate of healing, and a high relapse rate [1,2]. On the basis of histology and pathology, glioma is classified into four grades according to the World Health Organization (WHO) [3]. Glioblastoma multiforme (GBM, WHO grade IV) is the most aggressive and malignant subtype of glioma, whose median survival time is only 12–15 months. In spite of the advancements in surgery, as well as adjuvant therapy, the overall survival (OS) for individuals with glioma has changed slightly in the last decades [4]. Conventional treatments for glioma have not been sufficient to prevent tumor recurrence and progression. Therefore, it is urgent to develop new and more targeted therapies against glioma.

Cancers occur in intricate tissue environments, upon which they depend for sustained growth, infiltration, and metastasis [5]. There are increasing evidence that the tumor microenvironment (TME) plays an indispensable role in supporting the occurrence and progress of glioma [6]. The TME is composed of stromal cells, soluble factors, infiltrating immune cells, extracellular matrices, and other components. The TME in glioma tends to be immunosuppressive, which suggests immune dysregulation might play a role in glioma oncogenesis. Research documented that stromal cells play an imperative role in tumor growth, progression of disease, as well as drug resistance [7]. Additionally, stromal cells in the GBM environments could trigger human GBM cell growth by activating extracellular signaling. Moreover, infiltration of immune cells into tumors may contribute to the establishment of an immunosuppressive TME, which promotes tumor development, metastasis, and resistance to cancer therapies [8–10]. As an important component of TME, tumor-associated macrophages (TAMs) have been shown to create a favorable stroma for growth of tumors and numerous tumor-promoting activities, including angiogenesis, cell infiltration, and repression of adaptive anti-tumor immunity [11]. Increased infiltration of TAMs usually leads to tumor progression and poor prognosis for patients with glioma [12]. In recent years, targeted immunotherapy has received a great deal of attention. However, currently available immunotherapies for glioma have shown only a moderate clinical efficacy. Thus, it is of great clinical value to develop better immunotherapeutic strategies.

Rab small GTPases, which belong to the large Ras protein superfamily, are indispensable modulators of intracellular transport, as well as membrane trafficking in eukaryotic cells. The GTPases of the Rab family can modulate the specificity of the vesicular transport stages in cells. Along with their cross-talk proteins, Rab proteins would possibly constitute the intersection points between vesicular transport and metabolic signaling cascades, including growth factor signaling, glucose and lipid metabolism, as well as autophagy [13]. Increasing studies reported that Rab proteins have an indispensable role in the onset and progress of tumors [14,15]. Elevated expression of RAB23 enhanced cell migration along with infiltration of diffuse-type gastric cancer [16]. RAB25 expression was significantly up-regulated in ovarian and breast cancers, and accelerated the malignant progression of cancers by mediating AKT signaling and integrin recycling [17]. RAB7 was linked to poor outcomes of gastric cancer, as well as induced cancer cell proliferation, invasion, and migration by promoting the phosphorylation of PI3K and AKT [18]. In addition, accumulating evidence indicated that Rab proteins have core roles in immune response, differentiation of immune cells and the modulation of biological processes [19,20]. These findings imply that Rab proteins may serve as therapeutic targets for cancer.

RAB34, a Rab protein family member, is involved in repositioning of lysosomes, activation of micropinocytosis, and protein transport [21,22]. Recent investigations revealed that RAB34 participated in tumor oncogenesis and progression of multiple cancers. For example, RAB34 regulated cell adhesion, migration, and invasion by mediating endocytosis and recycling of integrin β3 in breast cancer [23]. Overexpression of RAB34 may lead to poor prognosis and tumor progression for patients with liver cancer [24]. Moreover, RAB34 promoted cell growth, migration along with tumorigenesis in non-small cell lung cancer [25]. However, the underlying function, as well as the mechanism of RAB34 in the progression of glioma remain unclear.

Herein, we assessed the expression and immune features of RAB34 in glioma at the molecular, as well as clinical levels via a large-scale analysis, and further investigated the biological role of RAB34 via in vitro experiments. We established that RAB34 was remarkably up-regulated in malignant glioma and was related to immune infiltration and tumor progression. A profound comprehension of the expression and feature of RAB34 would provide a molecular basis for the future immunotherapy of glioma.

## Materials and methods

### Data collection and human tissue samples

The TCGA cohort was abstracted from the University of California, Santa Cruz, Xena data resource (https://xenabrowser.net/), with a collection of 697 RNA-seq and related phenotypic data. An additional dataset including 325 RNA-seq data was obtained from the CGGA (Chinese Glioma Genome Atlas) data resource (http://www.cgga.org.cn/).

A total of 49 glioma tissues and 15 normal brain tissues were acquired from the Department of Neurosurgery, The Affiliated Wuxi No. 2 People’s Hospital of Nanjing Medical university, China. All samples had a confirmed pathological diagnosis as per the 2007 WHO classification by neuropathologists. Non-malignant brain tissue samples were acquired from 15 adult patients who were being treated with internal decompression surgery after experiencing a severe traumatic brain damage. The present study was approved by the Ethics Committee of The Affiliated Wuxi No. 2 People’s Hospital of Nanjing Medical University, and informed consent was obtained from all the patients.

### Cell culture

U251, U87, A172, and T98 glioma cells were obtained from the American Type Culture Collection (ATCC, U.S.A.). HA1800 normal human astrocyte cells were supplied by Boster Biological Technology, Ltd. (Wuhan, China). Cells were cultured in the DMEM medium (Invitrogen, Carlsbad, CA, U.S.A.) enriched with 10% FBS (Gibco, U.S.A.) along with 1% streptomycin-penicillin. All the cells were grown under 5% CO_2_ and 37°C conditions

### RNA isolation and quantitative real-time PCR (qRT-PCR)

The Trizol reagent (Invitrogen, U.S.A.) was employed to isolate total tissue and cellular RNA as described by the manufacturer. The quality along with the quantity of the RNAs were checked on the NanoDrop ND-2000 spectrophotometer (NanoDrop Technologies, Houston, TX, U.S.A.). After that, cDNA was generated with the PrimeScript™ RT Master Mix (TaKaRa Biotechnology, Dalian, China), Subsequently, qPCR was done with the QuantiFast SYBR Green PCR Kit (Qiagen, Germany).

### Western blot assay

The RIPA lysis buffer enriched with protease inhibitors (Beyotime Institute of Biotechnology) was employed to isolate total proteins from the cells and tissues, and span at 14000 ***g*** for 15 min at 4°C. Afterward, the BCA protein assay kit (Beyotime Institute of Biotechnology, Jiangsu, China) was employed to quantify the proteins. Thereafter, 40 µg of the proteins were fractionated on 12% SDS-PAGE gels. Then, the fractionated proteins were blotted on to 0.45 μm PVDF membranes (Thermo, U.S.A.), and 5% nonfat powdered milk in TBST was employed to block the membranes for 1 h. After that, the membranes were inoculated overnight with a primary antibody at 4°C. Thereafter, the membranes were rinsed thrice in TBST dried and incubated for 2 h with the respective HRP-labeled secondary antibody (1:5000). Visualization was performed by ECL (Absin, Shanghai, China), and the band intensity was detected by densitometry.

### Cell transfection

Small interfering RNA (siRNA) targeting RAB34 was chemically synthesized by GenePharma (Suzhou, China). Universal sequences were used as negative controls. Lipofectamine 3000 (Invitrogen, Carlsbad, U.S.A.) was used to transfect siRNAs into U251 and U87cells. All of the above procedures were performed as documented in the manual provided by the manufacturer.

### CCK-8 assay

The Cell Count Kit-8 was employed to explore cell proliferation as described by the manufacturer. After 2 days of transfection, 5000 cells/well were inoculated in 96-well plates for 24, 48, and 72 h. Next, 10 μl/well CCK-8 solution was introduced, followed by incubation for 2 h. The OD value of each sample was read on a spectrophotometer at 450 nm. The experiment was repeated thrice.

### EdU assay

The EdU assay was conducted using the 5-ethynyl-2′-deoxyuridine kit (C10310; Ribobio) according to the instruction manual. First, cells at a density of 2.5 × 10^5^/well were cultured in 96-well plates for 36 h. Subsequently, after incubating cells with 100 μl of 50 μM EdU for 2 h, cell fixative and 0.2% TritonX-100 were applied to fix cells for 30 min and permeabilize cells for 5 min at RT (room temperature), respectively. The cells then were inoculated with Apollo reaction mixture to mark the cells for 30 min. Ultimately, the nucleus was stained using Hoechst 33342 and visualized under a fluorescent microscope (Olympus).

### Transwell assay

Cell migration along with invasion abilities were tested by transwell assay. Cells were serum starved for 12–24 h before preparation of cell suspension to remove the serum. For migration assay, cells were resuspended in 200 μl serum-free medium and inoculated into the upper chamber. About 600 μl of DMEM with 10% FBS was then introduced into the lower chamber and the cells were incubated for 12 h. For invasion assay, Matrigel solution (BD, U.S.A.) was pre-coated in the upper chamber. Cells on the bottom surface were fixed with methanol and stained using 0.1% Crystal Violet. Five random fields per chamber were chosen and observed under an inverted microscope (Olympus). All experiments were replicated thrice.

### Statistical analysis

In the present study, the statistical analyses were mainly implemented in the R language. The R packages consisting of ggplot2, circlize, pheatmap, corrgram, corrplot, and pROC were employed to generate figures. For *in vitro* experiments, the statistical analysis was implemented in the GraphPad Prism V.7.0. Student’s *t* test or one-way analysis of variance was applied for analyzing the data. *P*<0.05 defined statistical significance.

## Results

### The expression of RAB34 is up-regulated in glioma

**Figure 1:**
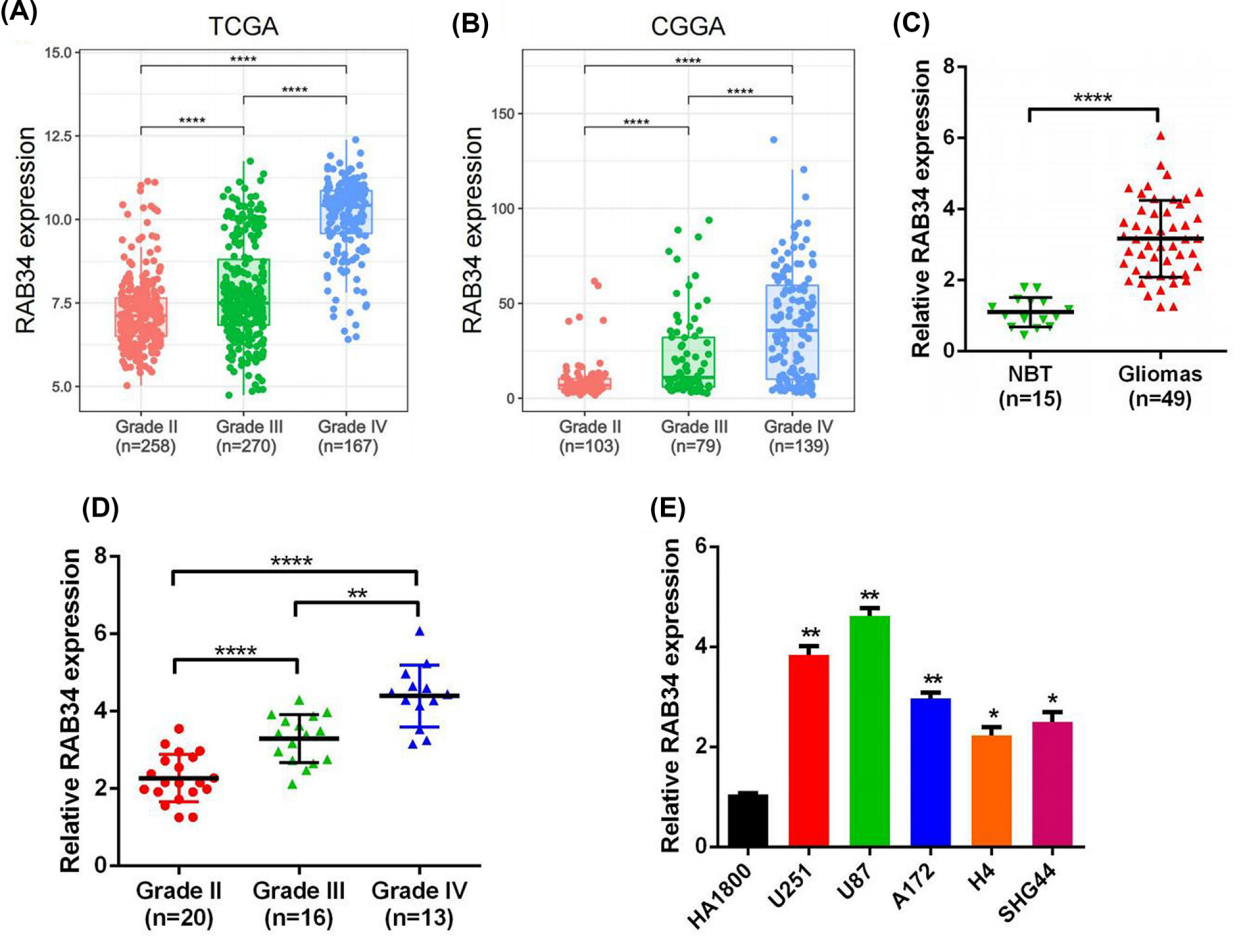
RAB34 expression is significantly increased in glioma (**A, B**) Comparison of RAB34 expression in TCGA and CGGA datasets with different WHO grades. (**C**) Validation of RAB34 mRNA expression in 49 glioma samples compared with that in 15 normal brain tissues (NBT) via qRT-PCR. (**D**) RAB34 mRNA expression in glioma of WHO grade II–IV was determined based on our patient samples. (**E**) The mRNA expression levels of RAB34 in different glioma cell lines and normal glial cell were determined by qRT-PCR; *P<0.05; **P<0.01; ****P<0.0001.

RAB34 expression data were abstracted from the TCGA and CGGA data resources. We found that RAB34 expression was remarkably higher in GBM (WHO IV) in contrast with that in WHO grade II, as well as grade III gliomas (Figure 1A,B). Besides, the increased expression, RAB34 was positively correlated with advanced WHO grade of glioma. To further confirm these results, we examined RAB34 expression level in glioma samples, as well as cell lines via qRT-PCR. The data illustrated that RAB34 expression was dramatically increased in glioma samples than that in normal brain tissues (Figure 1C). Furthermore, grade IV GBM samples showed the highest RAB34 expression, which is consistent with above results (Figure 1D). Moreover, RAB34 expression was up-regulated in U251, U87, A172, H4, and SHG44 glioma cells than that in HA1800 (Figure 1E). These results suggested that the abnormal expression of RAB34 might be involved in glioma progression.

### RAB34 expression is enriched in IDH wild-type glioma

**Figure 2:**
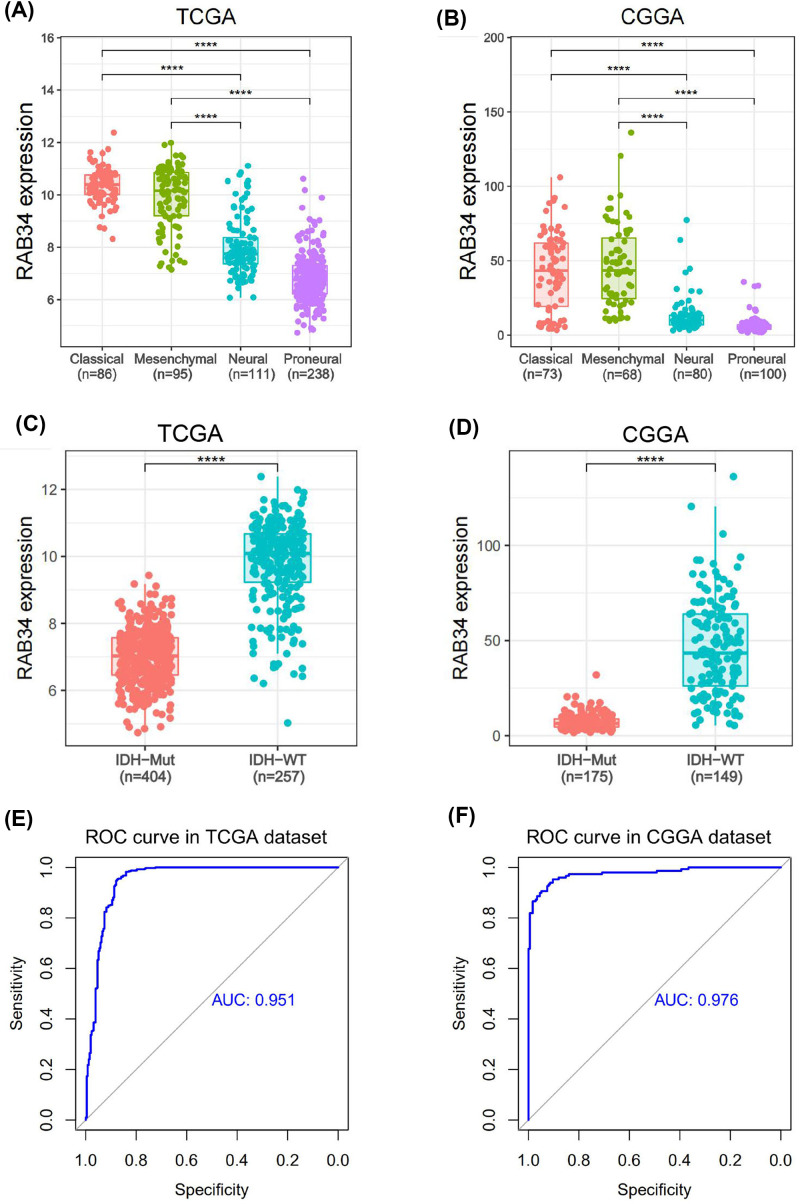
RAB34 expression is selectively distributed in different subtypes of glioma (**A,B**) RAB34 is overexpressed in classical and mesenchymal subtypes in TCGA and CGGA datasets. (**C,D**) RAB34 expression is significantly higher in IDH wild-type glioma than that in IDH mutant glioma in TCGA and CGGA datasets. (**E,F**) ROC curve analysis showed that RAB34 could serve as a signature for IDH wild-type glioma in TCGA and CGGA datasets; ****P<0.0001.

Next, we examined RAB34 distribution in four TCGA-defined molecular subtypes. The results revealed that RAB34 was apparently up-regulated in classical and mesenchymal subtypes than the other two subtypes in TCGA and CGGA datasets (Figure 2A,B). It is acknowledged that IDH mutation status is a clinically relevant molecular marker in glioma progression. Therefore, we further explored the relationship of RAB34 expression with IDH mutation status. We established that IDH wild-type glioma exhibited a higher content of RAB34 in contrast with IDH mutant glioma in TCGA and CGGA datasets (Figure 2C,D). Subsequently, receiver operating characteristic (ROC) analysis was employed to explore the diagnostic significance of RAB34 as a signature for diagnosing IDH status in glioma. Results showed that areas under curve (AUCs) were 95.1% and 97.6% in TCGA and CGGA cohorts, respectively (Figure 2E,F). These results suggested that RAB34 was selectively distributed and could serve as a signature for IDH wild-type glioma.

### RAB34 expression is negatively correlated with promoter DNA methylation

**Figure 3:**
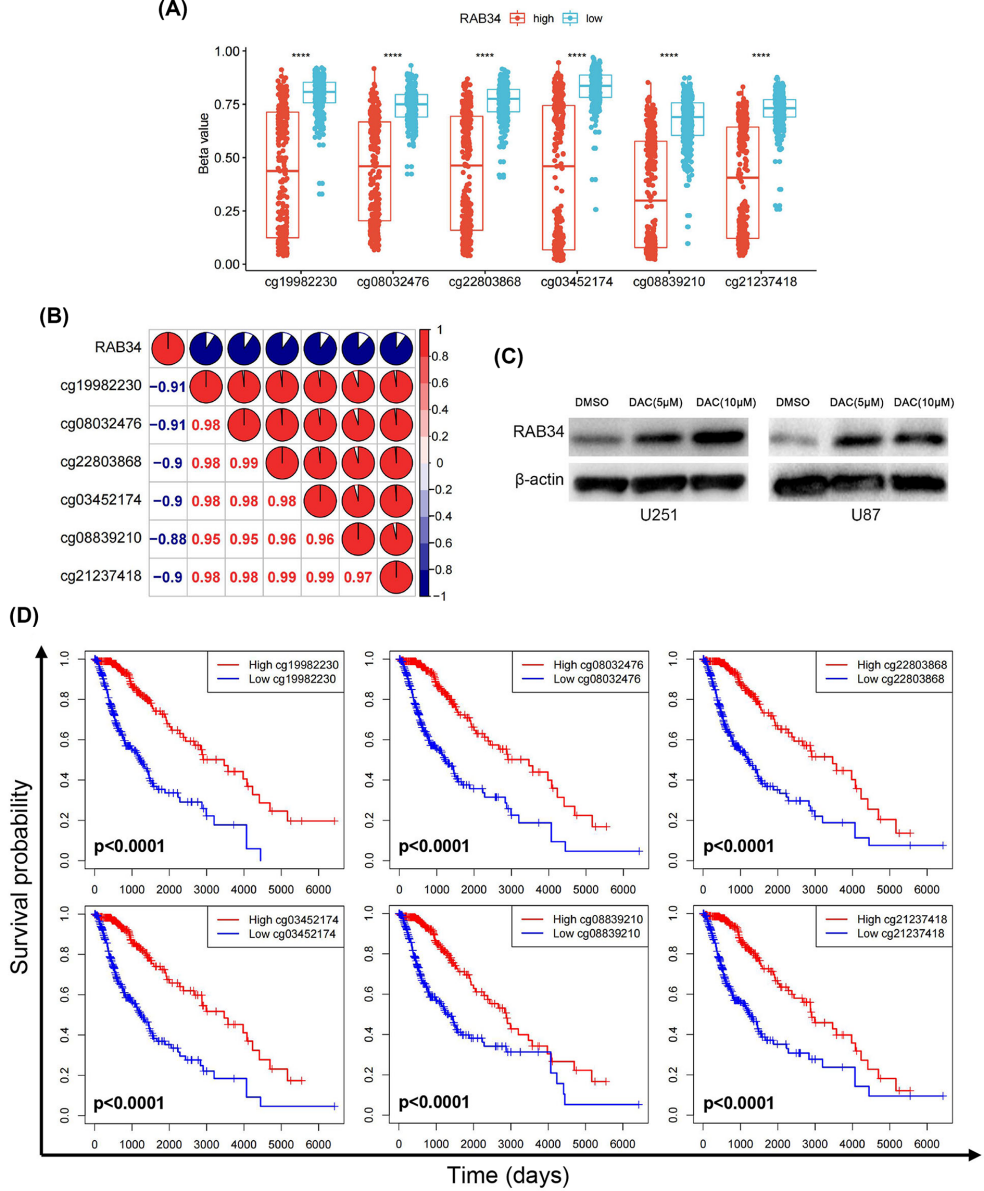
Promoter DNA methylation is correlated with RAB34 expression and glioma patients’ prognosis (**A**) Comparison of DNA methylation β-values at six CpG sites (cg19982230, cg08032476, cg22803868, cg03452174, cg08839210, and cg21237418) between low and high RAB34 expression groups. (**B**) Correlation between RAB34 expression and the promoter DNA methylation β-values in TCGA dataset. Results were displayed with the corrplot function from the corrplot package in R language. (**C**) Western blot results showed that RAB34 expression was increased in response to DAC (decitabine) in a dose of 5 or 10 μM in U251 and U87 cell lines. (**D**) The promoter DNA methylation levels of RAB34 was associated with overall survival time for glioma patients; ****P<0.0001.

To further investigate the possible mechanism of high RAB34 levels in glioma, we integrated TCGA glioma datasets and identified 594 patients with RAB34 expression along with DNA methylation data. In particular, six CpG sites (cg19982230, cg08032476, cg22803868, cg03452174, cg08839210, and cg21237418) were located in the promoter region of RAB34. Interestingly, we found that the DNA methylation β-values at all these six sites were markedly higher in LGG (lower grade glioma) than that in GBM (Supplementary Figure S1). Additionally, we divided the patient cohort into high- or low-expression group based on the median RAB34 expression value and found that the promoter DNA methylation β-values were higher in the low RAB34 expression group compared with that in the high RAB34 expression group (Figure 3A). Moreover, all the β-values at promoter region were negatively related to RAB34 mRNA expression (Figure 3B).

To further validate these results, we treated U251 and U87 cells with DAC (decitabine), which is a DNA methyltransferase inhibitor. Western blot results showed that RAB34 expression was increased in response to DAC in a dose of 5 or 10 μM compared with untreated cells (Figure 3C). Meanwhile, we found that patients who had low methylation levels exhibited a dramatically worse OS in contrast with those who had high methylation levels (Figure 3D). In summary, these data implied that RAB34 expression was possibly modulated by promoter DNA methylation, and this epigenetic modification could also serve as a promising prognostic signature for patients with glioma.

### RAB34 expression is related to immune functions and inflammatory activities in glioma

**Figure 4:**
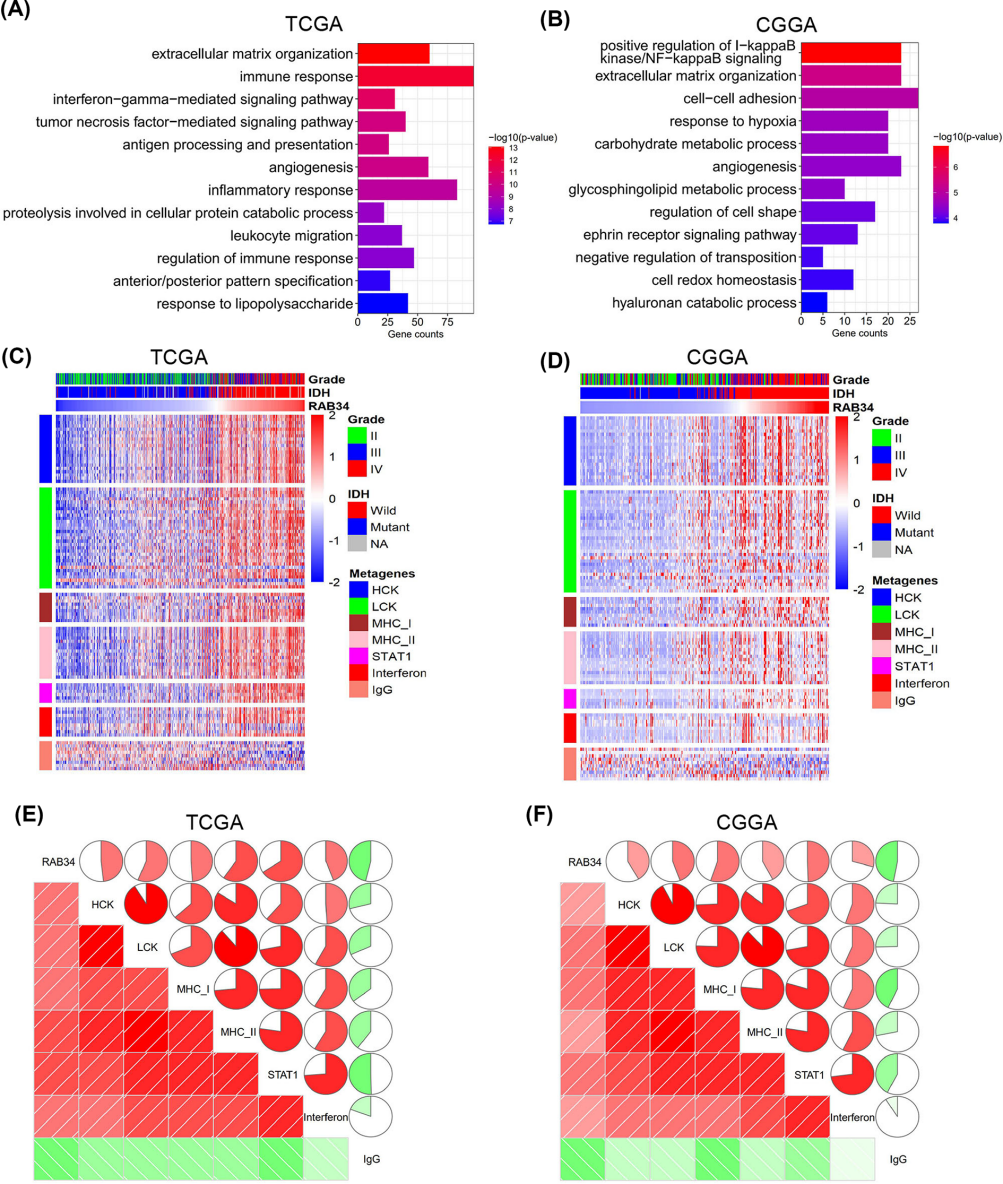
RAB34 expression is related to immune functions and inflammatory activities in glioma (**A**) GO analysis of the TCGA dataset showed that RAB34 was mainly involved in extracellular matrix organization, immune response, angiogenesis, and inflammatory response. (**B**) GO analysis of the CGGA dataset showed that RAB34 was mainly involved in positive modulation of I-κB kinase/NF-κB signaling, cell–cell adhesion, extracellular matrix organization, and response to hypoxia. (**C,D**) The heatmaps of RAB34-related clinicopathological parameters and inflammatory metagenes in TCGA and CGGA cohorts. (**E,F**) Corrgrams were established using the R package corrplot based on RAB34 expression and seven inflammatory metagenes. The results showed that RAB34 expression was positively related to HCK, LCK, MHC-I, MCH-II, STAT1, and interferon but negatively related to IgG in both TCGA and CGGA datasets.

To identify the biological effect of RAB34 in glioma, GO analysis was conducted. First, RAB34-linked genes in the cohorts were explored with Pearson’s correlation, with a cut-point of |*R*|>0.5 along with *P*<0.01. Overall, 2220 and 743 positive genes, as well as 1629 and 289 negative genes were detected as remarkably linked to expression of RAB34 in TCGA and CGGA cohorts, respectively. Then, we investigated the biological roles of these genes with the DAVID data resource. The results demonstrated that the genes positively correlated with RAB34 in TCGA dataset were enriched in extracellular matrix organization, immune response, angiogenesis, and inflammatory response (Figure 4A). Meanwhile, the positive genes in CGGA dataset were enriched in positive modulation of I-κB kinase/NF-κB signaling, cell–cell adhesion, extracellular matrix organization, and response to hypoxia (Figure 4B).

To further examine if RAB34 is associated with inflammatory response, seven clusters (including 104 genes) that were defined as metagenes were selected to represent diverse kinds of inflammation and immune response. RAB34 expression was positively linked to most of the clusters except for IgG in TCGA and CGGA datasets (Figure 4C,D). To verify these data, Gene Sets Variation Analysis (GSVA) was employed to convert these metagenes’ expression levels into enrichment scores, and then corrgrams were generated on the basis of the Pearson’s correlation values between RAB34 and seven metagenes. As illustrated in Figure 4E,F, in both TCGA and CGGA datasets, RAB34 expression was positively related to HCK, LCK, MHC-I, MCH-II, STAT1, and interferon, but negatively related to IgG, a marker for B cells. Taken together, these results indicated that RAB34 played an important role in the inflammatory response in glioma.

### RAB34 expression is correlated with stromal cells and immune cells in glioma

**Figure 5:**
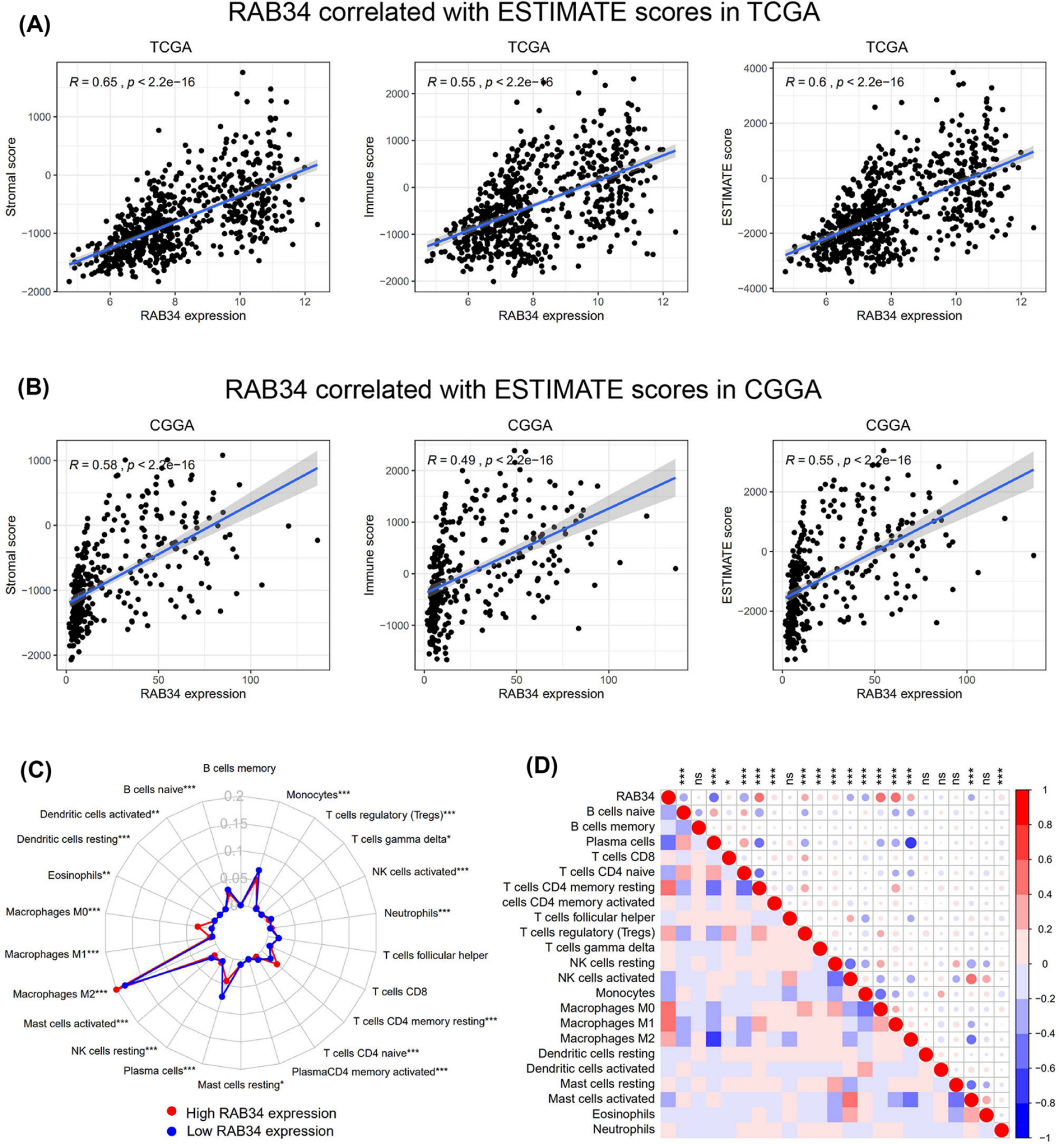
RAB34 expression is tightly associated with immune infiltration in glioma (**A,B**) RAB34 expression is positively correlated with immune score, stromal score, and ESTIMATE score in TCGA and CGGA datasets. (**C**) A comparative summary of the percentages of immune cells in different RAB34 expression groups estimated by CIBERSORT in TCGA dataset. (**D**) RAB34 expression is significantly correlated with infiltrated cells in the glioma microenvironment; ns, not significant, *P<0.05, **P<0.01, ***P<0.001.

Glioma tissues include not only glioma cells but also nontumor cells associated with gliomas, e.g., stromal cells along with immune cells. These nontumor cells dilute the purity of glioma cells and serve a vital role in glioma biology [26]. Previous researches reported that tumor purity was closely related to major clinical, as well as molecular characteristics, and low-purity cases were more likely to be diagnosed as malignant tumors and independently associated with shorter survival time [27]. Considering the GO analysis data, we next investigated whether RAB34 expression was linked to the infiltrated cells in glioma using ESTIMATE algorithm method described by Yoshihara [28]. The results demonstrated that RAB34 was remarkably related to the stromal score, immune score, and ESTIMATE score in both TCGA and CGGA datasets (Figure 5A,B). We further employed the CIBERSORT algorithm [29] to investigate the relative proportions of 22 subpopulations of infiltrating immune cells in glioma tissues from TCGA cohort. A comparative summary of the percentages of immune cells in different RAB34 expression groups was briefly depicted in Figure 5C. Notably, we identified that the percentages of M0 and M2 macrophages, and T cells CD4 memory resting were dramatically elevated in the high RAB34 expression group, while monocytes and plasma cells were remarkably enriched in the low RAB34 expression group. Next, we analyzed the relationship of RAB34 expression with 22 nonmalignant cell types. The results showed that ten types were positively related to RAB34 expression, whereas six types were negatively related (Figure 5D). These findings indicated that RAB34 may play a crucial role in the infiltration of stromal cells and immune cells in the glioma microenvironment.

### RAB34 expression is related to immune checkpoints in glioma

**Figure 6:**
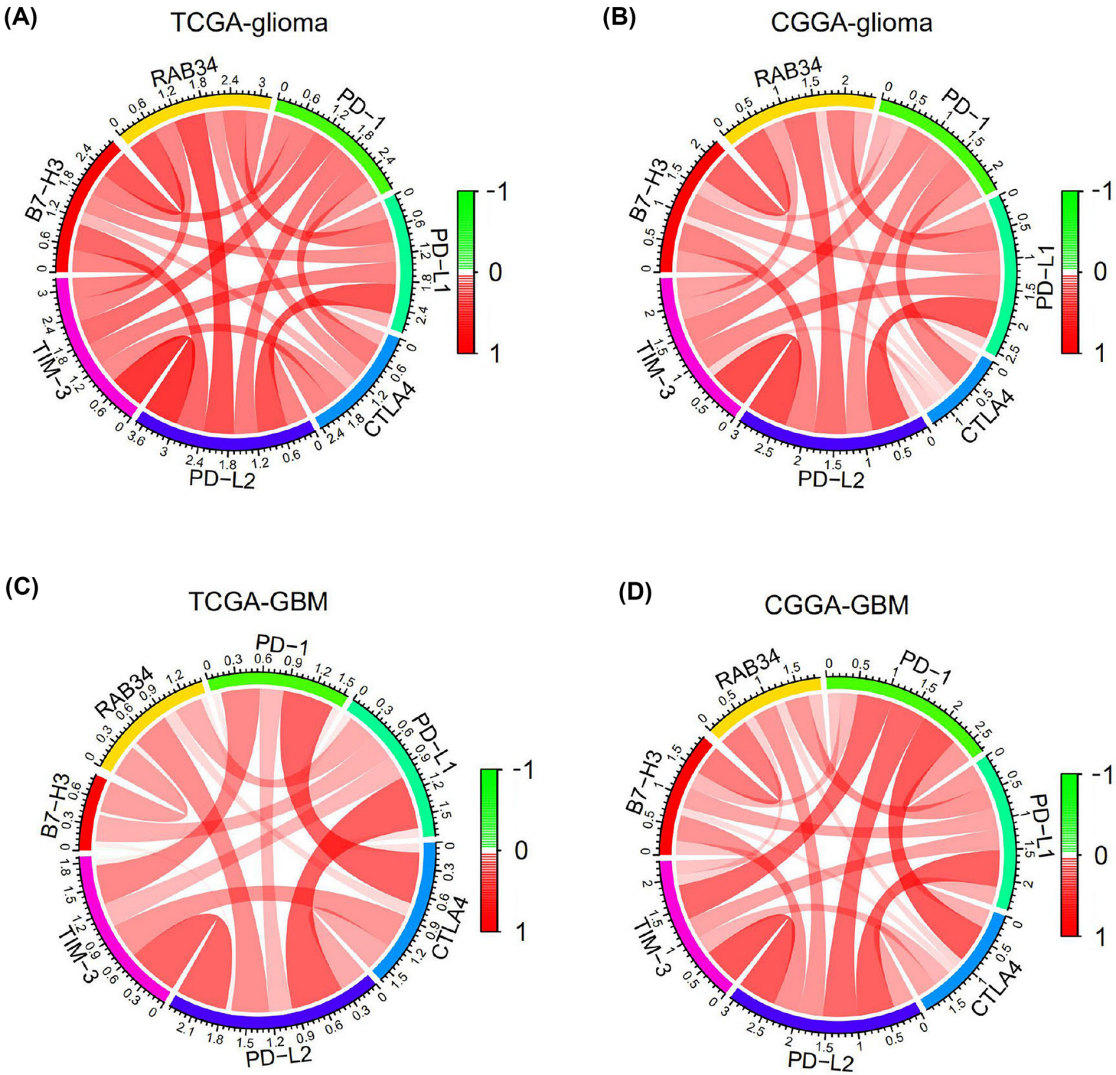
RAB34 expression is correlated with immune checkpoints (**A,B**) Correlation between RAB34 and immune checkpoints (PD-1, PD-L1, CTLA4, PD-L2, TIM-3, and B7-H3) in whole-grade gliomas in TCGA and CGGA datasets. (**C,D**) Correlation between RAB34 and immune checkpoints (PD-1, PD-L1, CTLA4, PD-L2, TIM-3, and B7-H3) in GBM in TCGA and CGGA datasets.

Immune checkpoint inhibitors designed to restore tumor-induced immunosuppression have become effective anticancer therapies [30]. Therefore, we performed Pearson’s correlation analysis using both TCGA and CGGA datasets to explore the relationship of RAB34 with immune checkpoints that have been investigated in clinical trials or clinical situations, including PD-1, PD-L1, CTLA4, PD-L2, TIM-3, and B7-H3. Intriguingly, RAB34 exhibited closely positive relationship with these immune checkpoints (PD-1, PD-L1, CTLA4, PD-L2, TIM-3, and B7-H3) in all grade gliomas in TCGA and CGGA cohorts as shown by Circos plots (Figure 6A,B). Similar findings were observed in GBM samples (Figure 6C,D). These results suggested the possible synergistic effects of RAB34 with these checkpoint members.

### Suppression of RAB34 inhibits EMT and the biological function of glioma cells

**Figure 7:**
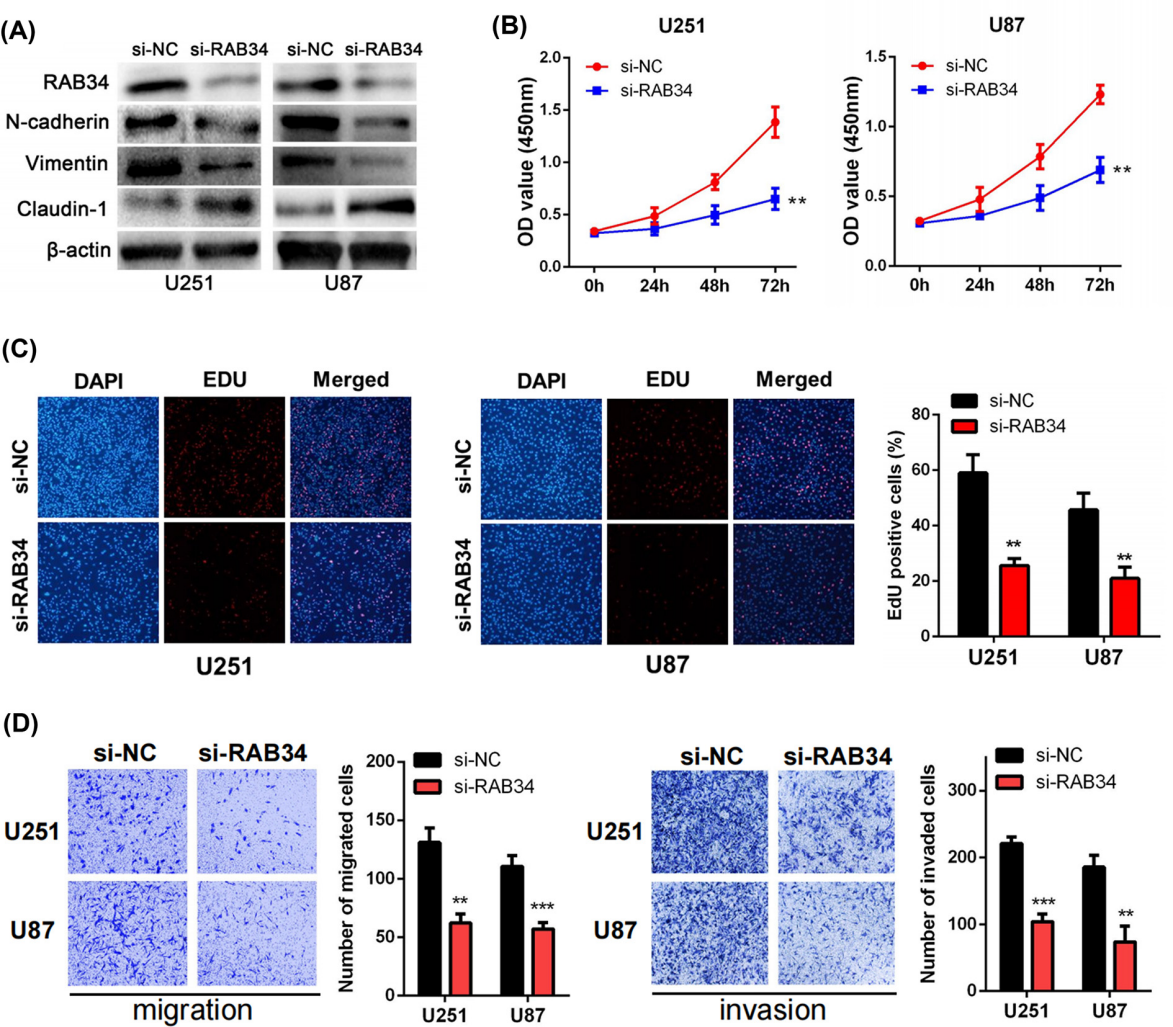
RAB34 promotes the EMT process, proliferation, migration, and invasion of glioma cells (**A**) Western blot assays were performed to detect the protein levels of RAB34, N-cadherin, Vimentin, and Claudin-1 after RAB34 knockdown in U251 and U87 cells. (**B**) CCK-8 assays showed that suppression of RAB34 inhibited cell viability in U251 and U87 cells. (**C**) EdU assays showed that suppression of RAB34 inhibited cell proliferation in U251 and U87 cells. (**D**) Transwell assays showed that reduction of RAB34 repressed the migration and invasion abilities of U251 and U87 cells, respectively; **P<0.01, ***P<0.001.

Firstly, we evaluated the relationship between RAB34 and EMT. Consistently, we found that RAB34 expression was dramatically linked to common EMT biomarkers based on TCGA and CGGA datasets (Supplementary Figure S2A and B). Furthermore, we conducted Western blot assays to assess the influence of RAB34 on the EMT process in glioma cells. As shown in Figure 7A, small interfering RNA si-RAB34 and negative control (si-NC) were inserted into U251 and U87 cells via transfection, respectively. si-RAB34 significantly reduced the expression of N-cadherin along with that of Vimentin but elevated the expression of Claudin-1 in U251 and U87 cell lines. In addition, CCK-8 assays showed that the viability of glioma cells was remarkably reduced after knockdown of RAB34 (Figure 7B). EdU assays also illustrated that the number of cells excited by red light in the si-RAB34 group was much less than that in the si-NC group (Figure 7C). In the transwell assays, we found that the reduction of RAB34 dramatically repressed the migration and invasion abilities of U251 and U87 cells (Figure 7D). These results revealed that RAB34 may contribute to the EMT progression of glioma and promote cell proliferation, migration, and invasion.

### Overexpression of RAB34 indicates worse prognosis for glioma patients

**Figure 8:**
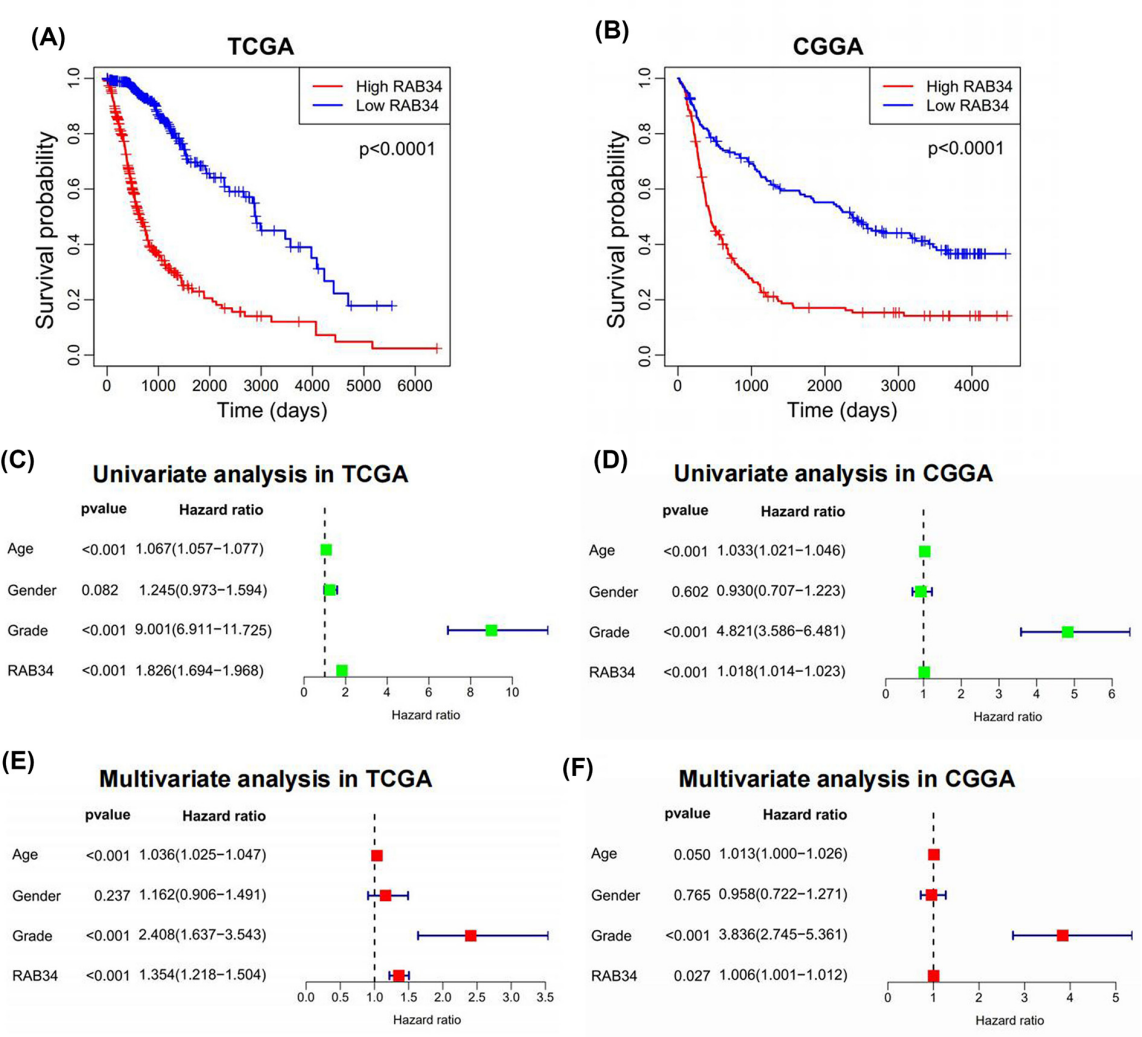
RAB34 predicts worse survival in glioma (**A,B**) Kaplan–Meier survival analyses showed that high RAB34 expression predicted poor prognosis for glioma patients based on the TCGA and CGGA datasets. (**C,D**) Univariate Cox regression analyses revealed that RAB34 was associated with overall survival of glioma patients in TCGA and CGGA datasets. (**E,F**) Multivariate Cox regression analyses revealed that RAB34 was an independent prognostic biomarker for glioma patients in TCGA and CGGA datasets.

Considering that RAB34 expression is abnormally expressed in glioma and correlates with histological grade, as well as molecular subtype, we speculated that RAB34 may be an indispensable prognostic signature for glioma patients. Thus, Kaplan–Meier curves were generated to explore its prognostic value. As illustrated in Figure 8A,B, patients with higher RAB34 expression had a dramatically shorter OS in contrast with patients with lower RAB34 expression in TCGA and CGGA cohorts. Simultaneously, we performed univariate and multivariate analyses to explore whether RAB34 could be an independent predictive factor for glioma patients. Univariate analysis indicated that RAB34 expression was remarkably correlated to OS (Figure 8C,D). In multivariate analysis, RAB34 expression was still a remarkable prognostic factor after correcting for other clinical factors, such as age, gender, and glioma grade (Figure 8E,F). In summary, RAB34 was an independent predictive factor for patients with glioma.

## Discussion

Glioma is the most common primary intracranial malignant tumor with a generally poor prognosis. Presenting extremely high mortality rates, GBM is the most aggressive subtype of brain tumor. Even with intensive therapies, consisting of neurosurgical resection, targeted treatment, adjuvant chemotherapy, as well as radiotherapy, most individuals with GBM experience relapse given the highly aggressive nature of this malignancy [31]. Immunotherapy, as one of the most potential strategies of activating antitumor immune response, has shown remarkable success for treating numerous cancers including glioma [32–34]. However, low response rate and treatment resistance originating from the precise interaction of cytokines with immune cells severely limited the widespread application of immunotherapy [35]. At present, one promising treatment approach is to design immunotherapy against glioma through targeting the highly expressed proteins that have an indispensable role in immune repression. Therefore, it is urgently needed to evaluate novel targets of immunotherapy.

RAB34, a type of RAB proteins, has been documented to be expressed abnormally and play critical roles in diverse kinds of cancer. However, its function remained unclear in glioma. Herein, we comprehensively analyzed the oncogenic role of RAB34 in the malignant progress of glioma by conducting a large-scale analysis and *in vitro* experiments. The data demonstrated that the expression of RAB34 was not only aberrantly overexpressed in glioma but also dramatically correlated with WHO grade. IDH wild-type glioma is recognized generally to have a worse OS in contrast with IDH mutant glioma. Our results illustrated that RAB34 was highly enriched in IDH wild-type glioma. In addition, high RAB34 expression predicted a worse prognosis, which is consistent with its enrichment in IDH wild-type glioma. Furthermore, our research certified that RAB34 was an independent predictive marker for individuals with glioma. Accumulating studies have shown that DNA methylation, the primary epigenetic modification, participates in the initiation and progression of different tumors. Moreover, aberrantly methylated promoters have been identified as prospective biomarkers for the diagnosis and prognosis of glioma [36,37]. Therefore, we explored the potential mechanism of RAB34 overexpression and found that RAB34 expression was significantly negatively related to the promoter methylation levels. Additionally, the promoter methylation levels of RAB34 in GBM was significantly lower than that in LGG. Furthermore, we verified that the expression of RAB34 was significantly increased after treatment with DAC in glioma cell lines. Our results indicated that hypomethylation of RAB34 promoter region may be a key contributor to the increased RAB34 expression in glioma. However, our study only demonstrated a correlation between RAB34 expression and DNA methylation, more in-depth experiments are needed to verify whether the increased expression of RAB34 is directly caused by DNA methylation in glioma.

Immunotherapy is becoming the most potential therapy for malignant tumors. Nevertheless, the TME, composed of extracellular matrix, stromal cells, vasculature, and inflammatory cells, is often involved in supporting the progress of glioma. Therefore, it is of great importance to identify novel molecular biomarkers, as well as targets that make an important impact in the TME. Herein, we established that RAB34 played an indispensable role in inflammatory response, immune response, and angiogenesis, as well as remarkably positively correlated with infiltrated stromal cells along with immune cells. The immune escape mechanism of tumorigenesis has been demonstrated by numerous studies, and a number of immunosuppressive agents have been applied clinically in recent years. Immune checkpoint inhibitors in particular have demonstrated remarkable success in treating many tumors [38,39]. For instance, PD-1 and PD-L1 inhibitors showed significant improvement in melanoma, non-small cell lung cancer, kidney cancer, and other solid malignancies [40,41]. Monoclonal antibodies directed against CTLA4, such as ipilimumab, presented significant clinical benefit for individuals with metastatic melanoma [42]. However, due to the side effects of autoimmunity, it is difficult for immunotherapy to be widely used. Accumulating research is focusing on studying a combination of immune checkpoint blockades, which may provide a potential therapy for glioma [43–45]. Given the remarkable efficacy of immune checkpoint blockade treatment, we demonstrated that RAB34 expression was tightly associated with the checkpoint proteins PD-1, TIM-3, PD-L1, B7-H3, PD-L2, and CTLA4, and demonstrating the promising impact of RAB34 on modulating these immune checkpoints.

Recently, increasing research evidence has illustrated that the cross-talk of EMT-related factors with the TME could facilitate tumor immune escape, indicating that EMT could play a key role in tumor immunosuppression and immune evasion [46,47]. In addition, previous studies have shown that EMT is linked to the activation of various immune checkpoint molecules such as PD-L1 [48]. EMT-triggered immune evasion enhances the progress of cancer and might also provide a platform for the discovery of new therapies and biomarkers for predicting response to checkpoint inhibitors. Herein, we found that RAB34 expression was remarkably related to EMT markers and promoted EMT process in glioma. Furthermore, RAB34 promoted the proliferation, migration, and infiltration of glioma cells. These findings indicated that RAB34 may play a pro-cancer role in glioma.

In conclusion, this study discovered a novel function for RAB34 in enhancing the malignant phenotype and resulting in immune evasion of glioma. Our findings highlighted the potential of RAB34 as a novel immunotherapeutic target for glioma.

## Supplementary Material

Supplementary Figures S1-S2

## Data Availability

Publicly available datasets were analyzed in this study. TCGA dataset can be found at: https://xenabrowser.net/datapages/?cohort=TCGA%20lower%20grade%20glioma%20and%20glioblastoma%20(GBMLGG)&removeHub$=$https%3A%2F%2Fxena.treehouse.gi.ucsc.edu%3A443,GSE16011; CGGA dataset can be found at http://www.cgga.org.cn/download.jsp.

## Abbreviations

AUC: area under curve
CGGA: Chinese Glioma Genome Atlas
DAC: decitabine
DAVID: The Database for Annotation Visualization and Integrated Discovery
EMT: epithelial-to-mesenchymal transition
GBM: glioblastoma multiforme
GO: gene ontology
GSVA: Gene Sets Variation Analysis
IDH: isolated diastolic hypertension
LGG: lower grade glioma
OS: overall survival
ROC: receiver operating characteristic
TAM: tumor-associated macrophage
TCGA: The Cancer Genome Atlas
TME: tumor microenvironment
