# Supplementary material for: Overexpression of RAB34 associates with tumor aggressiveness and immune infiltration in glioma
Source: Biosci Rep. 2022 Oct 28;42(10):BSR20212624. doi: 10.1042/BSR20212624 (PMC9620491; doi:10.1042/BSR20212624)
Supplement: Supplementary Figures S1-S2 [file BSR-2021-2624_supp.pdf]

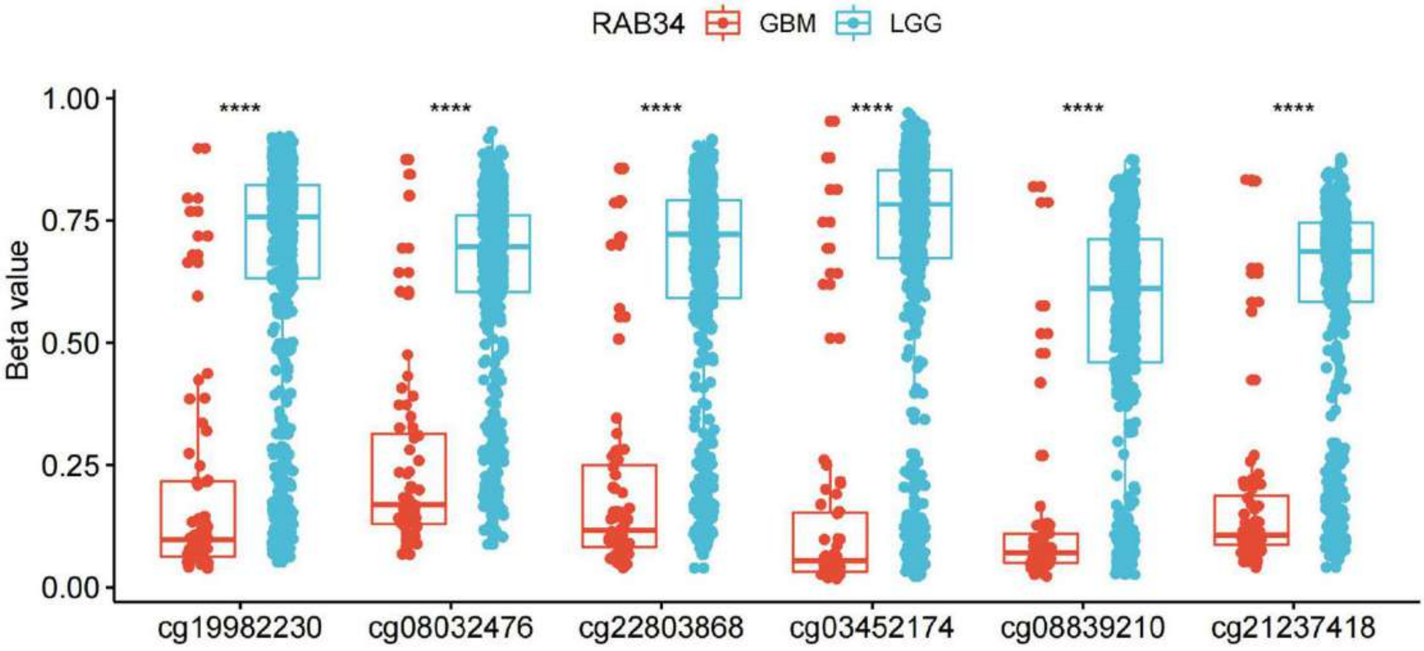
RAB34
GBM
LGG
1.00
0.75
0.50
0.25
0.00
Beta value
****
cg19982230
cg08032476
cg22803868
cg03452174
cg08839210
cg21237418

Figure S1. The DNA methylation beta values at six CpG sites (cg19982230, cg08032476, cg22803868, cg03452174, cg08839210, and cg21237418) were markedly higher in LGG (lower grade glioma) than that in GBM in TCGA dataset. **** $P < 0.0001$.

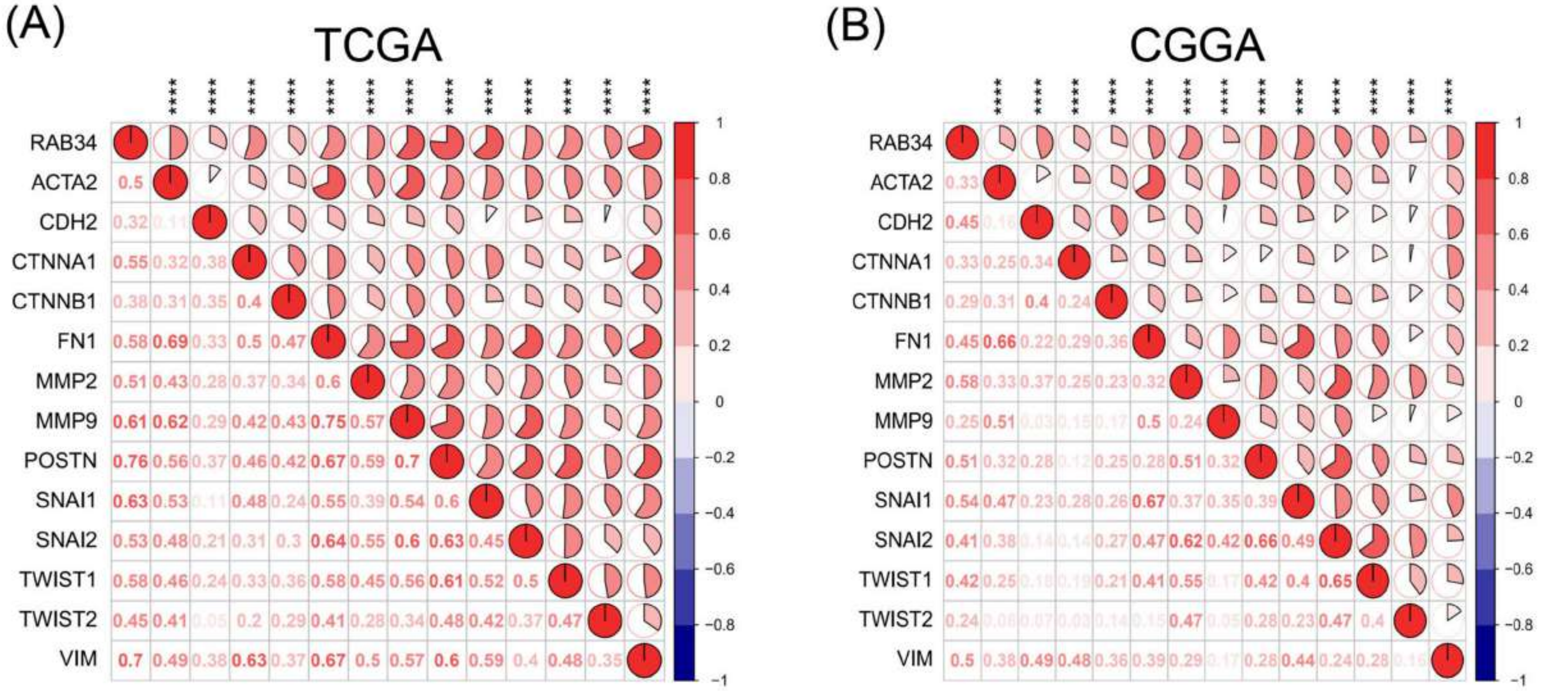
(A)
TCGA
(B)
CGGA
RAB34
ACTA2
CDH2
CTNNA1
CTNNB1
FN1
MMP2
MMP9
POSTN
SNAI1
SNAI2
TWIST1
TWIST2
VIM
1
0.8
0.6
0.4
0.2
0
−0.2
−0.4
−0.6
−0.8
−1

Figure S2. The relationship between RAB34 expression and common EMT biomarkers. **A, B** RAB34 expression was dramatically linked to common EMT biomarkers based on TCGA and CGGA datasets. **** $P < 0.0001$.
